# A Bibliometric Analysis of Electrospun Nanofibers for Dentistry

**DOI:** 10.3390/jfb13030090

**Published:** 2022-07-09

**Authors:** Shixin Jin, Andy Wai Kan Yeung, Chengfei Zhang, James Kit-Hon Tsoi

**Affiliations:** 1 Dental Materials Science, Division of Applied Oral Sciences and Community Dental Care, Faculty of Dentistry, The University of Hong Kong, Hong Kong, China; jasonjin@connect.hku.hk; 2 Oral and Maxillofacial Radiology, Division of Applied Oral Sciences and Community Dental Care, Faculty of Dentistry, The University of Hong Kong, Hong Kong, China; ndyeung@hku.hk; 3 Restorative Dental Sciences, Faculty of Dentistry, The University of Hong Kong, Hong Kong, China; zhangcf@hku.hk

**Keywords:** electrospun nanofibers, dentistry, bibliometric

## Abstract

Electrospun nanofibers have been widely used in dentistry due to their excellent properties, such as high surface area and high porosity, this bibliometric study aimed to review the application fields, research status, and development trends of electrospun nanofibers in different fields of dentistry in recent years. All of the data were obtained from the Web of Science from 2004 to 2021. Origin, Microsoft Excel, VOSviewer, and Carrot^2^ were used to process, analyze, and evaluate the publication year, countries/region, affiliations, authors, citations, keywords, and journal data. After being refined by the year of publication, document types and research fields, a total of 378 publications were included in this study, and an increasing number of publications was evident. Through linear regression calculations, it is predicted that the number of published articles in 2022 will be 66. The most published journal about electrospun dental materials is Materials Science & Engineering C-Materials for Biological Applications, among the six core journals identified, the percent of journals with Journal Citation Reports (JCR) Q1 was 60%. A total of 17.60% of the publications originated from China, and the most productive institution was the University of Sheffield. Among all the 1949 authors, the most productive author was Marco C. Bottino. Most electrospun dental nanofibers are used in periodontal regeneration, and Polycaprolactone (PCL) is the most frequently used material in all studies. With the global upsurge in research on electrospun dental materials, bone regeneration, tissue regeneration, and cell differentiation and proliferation will still be the research hotspots of electrospun dental materials in recent years. Extensive collaboration and citations among authors, institutions and countries will also reach a new level.

## 1. Introduction

Since their discovery, nanomaterials have been widely used in many fields, such as environmental protection, energy harvest and storage, aerospace, electronics, catalysis, photonics, personal protection, biomedical science, and textile and clothing [1,2,3,4,5,6,7,8,9,10,11]. The application of nanomaterials in dentistry was first proposed by Freitas Jr, R. A. in 2000 and has developed rapidly since then [12]. In the beginning, nanomaterials were used as nanoparticles in toothpaste, mouthwashes, composite resins, and adhesives [13,14,15,16]. As the technology develops, more nanomaterials have been used in different aspects of dentistry, including disease testing, cell differentiation induction, and dental implants [17,18,19,20]. Among the many types of nanomaterials, nanofibers played a significant role due to their high porosity and specific surface area.

Phase separation, drawing, template synthesis, self-assembly and electrospinning can be used for nanofiber fabrication. Among these methods, electrospinning has been widely used in many fields since the 1990s as a technology for simple and effective production of nanofibers [21,22]. There are many materials suitable for electrospinning, which can be organic or inorganic, natural polymers or synthetic polymers, and the product of electrospinning can be fiber, membrane, and scaffold; it can even contain some designed patterns [23,24,25].

**Figure 1:**
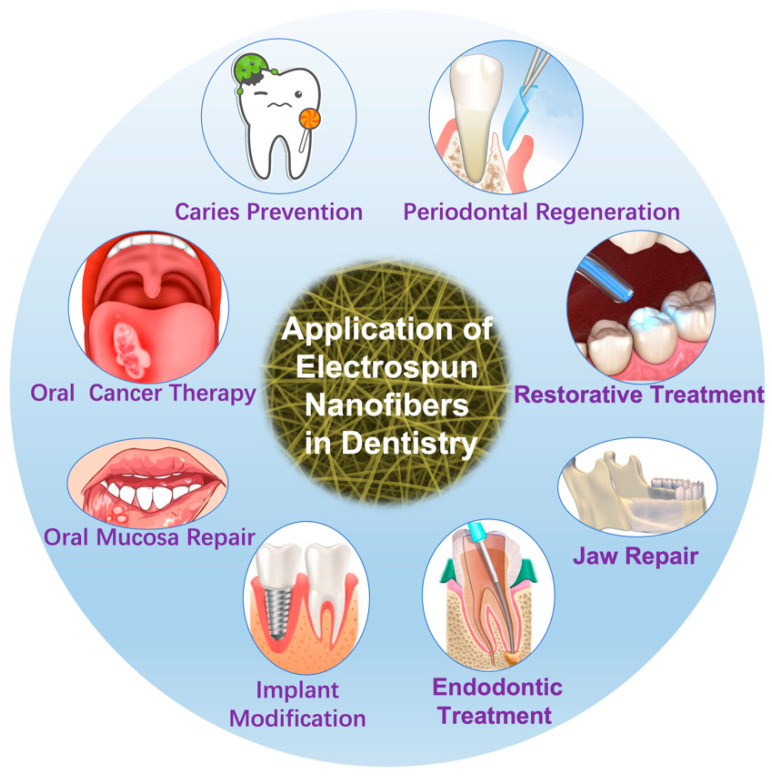
Application of electrospun nanofibers in dentistry.

In 2004, electrospun nanofiber was first used for Bisphenol A-glycidyl methacrylate/Triethylene glycol dimethacrylate (BIS-GMA/TEGDMA) dental restorative composite resins reinforcement [26]. After more than ten years of exploration and development, electrospun nanofibers are now used in tissue regeneration, restoration, and drug delivery, as illustrated in Figure 1.

Bibliometric analysis has been widely used to summarize research trends for detecting the state for a particular research field [27,28,29]. In this paper, bibliometric analysis was employed to investigate the application of electrospun nanofibers in dentistry. The principle of electrospinning and the materials suitable for electrospun dental nanofibers were briefly introduced. The application fields in dentistry of electrospun nanofibers were systematically described in the literature review. In addition, the obtained data were analyzed by publish year, journal, author country/region, author institute, author citation, and keywords; the cooperation was also studied by the network map.

## 2. Literature Review

### 2.1. Technology for Electrospinning

Research on electrospinning can be traced back to the early 20th century, but the popularity of electrospinning to produce nanofibers was not promoted until after the 1990s, and the number of related papers, patents, and books published in the following two decades has been increasing exponentially [30,31,32,33]. Nowadays, due to the unique properties of electrospun nanofibers, and the rapid development of modern electrospinning technology, electrospun nanofibers have been used in various industries.

#### 2.1.1. Principle of Electrospinning

The basic setups of electrospinning include a high voltage power supply, a syringe pump, a spinneret, and a collector [34]. When the high voltage was applied to the spinneret, a Taylor cone will be produced, and then charged jet will be ejected after the surface tension was overcome, a straight line extends near the spinneret, and then the jet flies to the collector in a spiral manner under the stretch of electrostatic force [35,36]. The electrospinning device can be separated into solution electrospinning and melt electrospinning [37,38]. For solution electrospinning, many different natural and synthetic polymers have been successfully made, and even some inorganic nanofiber can be made using well-designed precursor and post-processing processes [39,40]. As to melt electrospinning, it is mainly used for polymers which are difficult to dissolve in appropriate solvent [25].

#### 2.1.2. Parameters Affecting Electrospinning

Many parameters can affect the process, the nanofiber’s morphology, and property during the electrospinning. The parameters mainly include the following: polymer parameters (such as molecular weight, conductivity, viscosity, concentration, and solvent system) [41,42,43], processing parameters (such as the voltage, distance, spinneret, collector, and flow rate) [44,45,46,47,48,49], and environment parameters (such as temperature and humidity) [50].

The polymer property determines whether it can be used for electrospinning; for example, if there is no conductivity or the conductivity of the polymer is very low, the electric field force could not stretch the polymer into fibers [51,52]. During the processing, the electric field intensity was mainly determined by the applied voltage, and the electric field distribution was mainly affected by the receiving distance, the shape of the spinneret and collector [53,54]. As to the temperature and humidity, the main influence is the temperature of the fluid and the evaporation rate of the solvent [55,56].

### 2.2. Materials for Electrospinning in Dentistry

For dental materials, it is best to be functional and biocompatible [57,58]. Dental materials suitable for electrospinning mainly include natural polymers (such as collagen, chitosan, gelatin, and silk fibroin), synthetic polymers (such as Polyvinyl alcohol, Poly-L-lactic acid, and Polycaprolactone), and nanocomposites (such as hydroxyapatite blends). Besides, some metals and metal oxides (such as silver and titanium oxide, and zinc oxide) and drugs (such as chlorhexidine and ampicillin) can be added to electrospun dental nanofibers as functional particles. Table 1 shows the materials which can be used as the main body for electrospun dental nanofibers.

### 2.3. Applications of Electrospinning in Dentistry

The significant applications of electrospun nanofibers include periodontal regeneration, restorative treatment, endodontic treatment, implant modification, mandibular repair, oral mucosa repair, oral cancer therapy, and caries prevention. Here, some significant applications were summarized in the following sections.

#### 2.3.1. Periodontal Regeneration

The periodontium consists of gingiva, periodontal ligament (PDL), cementum, and alveolar bone [76]. Periodontal disease is triggered by the accumulation of plaque biofilm at the gingival–tooth interface, which eventually extends into the subgingival niche, leading to the destruction of periodontal tissue and ultimately tooth loss, and affects about 15% of the adult in the world [77,78]. Besides, the trauma and tumor resection can also lead to the defects of periodontal tissues [79,80]. Guided tissue regeneration (GTR) and guided bone regeneration (GBR) are the two effective methods of regenerating periodontal tissues [81,82]. Most of the membrane made of natural or synthetic polymer has good biocompatibility, biodegradability, osteoconduction, and osteoinductivity.

Electrospun nanofiber mats loaded with drugs can be used as drug delivery systems to reduce the depth of periodontal pockets and improve patients’ oral condition with chronic periodontal disease, drugs such as metronidazole and chlorhexidine have been successfully employed as antibacterial agents added to the nanofiber mat [83,84,85]. This method is simple and effective, and it can be used as a new idea for the treatment of periodontitis.

The periodontal ligament, cementum, and alveolar bone defects can also be regenerated by GBR/GTR membrane [86,87]. When performing the defect regeneration, it can act as a barrier membrane preventing fibroblasts from entering the bone defect site and promoting osteoblast adhesion, proliferation and bone tissue regeneration, and the use of the degradable GBR/GTR membrane can reduce the likelihood of post-surgical bacterial infection and possible damage caused by additional surgery [88,89]. In addition to the functions mentioned above, the introduction of additives (such as growth factors, drugs, proteins, bioactive ceramics, and metal oxide) can provide more possibilities for its application. For example, the PCL electrospun fibrous membrane doped with 0.5% (*w*/*v*) Zinc oxide (ZnO) exhibits excellent osteoconductivity and antibacterial properties in rat periodontal defect experiments [90]. The assembled functionally graded membrane loaded with platelet-derived growth factor (PDGF)-metronidazole can effectively promote alveolar ridge regeneration, prevent wound dehiscence, and accelerate the regeneration process [91].

#### 2.3.2. Restorative Treatment

Resin-based composites are the most popular dental restoration materials, in order to improve the strength, impact resistance and fatigue resistance property and reduce the damage caused by insufficient strength during the utilization [92,93], fillers were used to enhance the mechanical properties of resin-based composites, electrospun nanofiber was employed as a type of nanofiller [94]. Electrospun nylon 6 nanofiber was first used to reinforce the Bis-GMA/TEGDMA dental restorative composite resins. The addition of 5% (mass fraction) nanofibers can improve the flexural strength, elastic modulus, and work of fracture by more than 20% [26]. SiO_2_, Caron nanotube, glass fiber, etc., have been successfully used to reinforce resin-based dental composites [95,96,97]. In addition, the effect of fiber orientation on composite properties was also investigated, it was reported that the flexural properties of Bis-GMA dental restorative composites reinforced with post-drawn polyacrylonitrile-poly(methyl methacrylate) (PAN-PMMA) nanofiber were further increased in comparison with that nanofiber without treatment [98].

#### 2.3.3. Endodontic Treatment

Endodontic treatment can preserve the function of the pulp to a certain extent and allows for continued root development, and this will give patients with endodontics an option other than root canal treatment or tooth extraction [99,100]. Electrospun scaffolds have been used for pulp regeneration and exhibited good inducibility. Bottino et al. produced many electrospun scaffolds with different drugs, the 3D porous scaffolds not only provide support for the attachment and proliferation of human pulp-derived cells, but also deliver drugs to the root canal and pulp cavity [101,102]. It was reported that halloysite nanotubes (HNTs), Metronidazole (MET), Ciprofloxacin/(CIP), etc., were added to the polydioxanone (PDS), and the antibacterial property, regenerative property can be improved significantly. Apatite-mineralized electrospun polycaprolactone nanofiber scaffolds were also investigated, and evaluations of cell adhesion, growth, and odontoblast differentiation showed the positive effects of the scaffolds were considered to be promising for dental tissue engineering [103]. In addition, the incorporation of growth factors (such as nerve growth factor) can significantly induce pulp regeneration [104].

#### 2.3.4. Implant Modification

The metal implant has disadvantages, including difficulty achieving sufficient chemical bonding with the surrounding bone, especially in the early stage of implantation, because the implants are inherently biologically inert [105]. In order to promote osteogenesis, osteoconduction and osseointegration, modifications on the surface of the titanium implants are still necessary [106,107], and fabrication of porous implant surface and coating are the most studied methods [106,108]. As a simple technology to prepare nanofibrous structure materials, electrospinning has been used to modify the implant surface in recent years, electrospun piezoelectric membrane, as graft has been reported in orthopedic implants and heal injured cartilage recently [109,110,111]. The dental implant surface coated with PLGA/collagen fibers showed higher cell adhesion and cell proliferation rates and favored higher cell proliferation, differentiation, and mineralization [112]. Antibacterial dental implant coating could also be obtained by electrospinning technology, and it was reported that PLA: PCL/GEL nanofibers containing tetracycline hydrochloride (TCH) enable titanium dental implants to be simultaneously antimicrobial and osteoinductive [113,114]. In addition to the surface modification of metal implants, electrospun nanofibers can also be used to modify denture resins [64].

#### 2.3.5. Jaw Repair

Jaw defects can result in periodontitis, tumor, trauma, and congenital diseases, etc. [115]. The patients often have to bear the double burden of physical and psychological damage. Although autologous bone graft is still the gold standard technique for bone filling, a large number of alternative bone grafts are still required each year [79,116]. The development of biomedical materials has brought new treatment options for jaw defects. Among them, electrospun nanofiber materials have been widely used in the research of jaw defect repair. Three-dimensional fibrous SiO_2_ nanofibers with chitosan as bonding sites (SiO_2_ NF-CS) scaffolds were developed by electrospinning and lyophilization technique, the in vivo experiments demonstrated that SiO_2_ NF-CS scaffolds could fill the mandibular defect in rabbits [117]. The poly-caprolactone/nano-hydroxyapatite/beta-calcium phosphate (PCL/nHA/β-TCP) composite scaffolds loaded with poly-(lactic-co-glycolic acid)/nano-hydroxyapatite/collagen/heparin sodium (PLGA/nHA/Col/HS) small vascular stents were sufficient to repair critical defects, which were more than 25 mm in rabbit mandibles [118]. Biocompatible and osteoinductive electrospun nanomaterials have shown great potential in jaw defect repair.

#### 2.3.6. Oral Mucosa Repair

Oral mucosa plays a critical role in oral cavity surface protection. However, oral mucosal disease is a very common oral disease, and trauma, maxillofacial cancer, and congenital cleft lip and palate can also cause certain oral mucosal defects [119]. Electrospun nanomaterials have been widely used for drug delivery and wound dressing due to the large specific surface area, controllable porosity, good ductility, etc. [120,121]. As a natural polymer with good cytocompatibility, silk fibroin is very suitable for tissue engineering [122,123,124]. Gelatin, PLGA, etc., have been reported to prepare electrospun membranes for oral mucosa regeneration [70,125,126]. The silk fibroin electrospun matrix can promote the mucosa wound healing, in addition, the electrospun silk fibroin membranes grafted with surface-aminated liposomes (NH_2_-LIPs) and polydopamine (PDA) via conjugation could stimulate blood vessel regeneration and facilitate the recovery of damaged oral mucosa [127,128]. In another study, chitosan-coated Eudragit/Human Growth Hormone (hGH) nanofibrous sheets were prepared by electrospinning and dip-coating for oral mucositis, sheets incorporating hGH significantly increased the proliferation of human dermal fibroblasts, and in vivo studies in dogs showed that chitosan-layered sheets show accelerated wound recovery [129]. In addition to dip-coating techniques, magnetron sputtering and plasma treatment have also been applied to electrospun nanofiber material modification to prepare biomaterials for oral mucosal regeneration [130].

#### 2.3.7. Oral Cancer Therapy

Oral cancer is the sixth-most common cancer in the world [131,132]. As a novel strategy for oral cancer therapy, nanomedicine has been studied for the past decades [133,134]. Electrospinning plays a significant role in nano-drug delivery systems for cancer therapy [135,136]. In a study, electrospun composite membranes were made with Angelica gigas Nakai (AGN) extract, PVA and Soluplus, the AGN-loaded nanofiber structure could be dissolved and absorbed very fast, it exhibited excellent antiproliferation property against the proliferation of oral squamous cell carcinoma cells [71], similarly, Phloretin and d-α-tocopheryl polyethylene glycol succinate (TPGS) were also used by this group in the treatment of oral squamous cell carcinoma, which was encapsulated in nanofibers by electrospinning [137]. Another study reported that the anti-cancer drug 5-Fluorouracil (5-FU) was incorporated into the PLLA scaffold to treat oral cancer [138]. In conclusion, electrospun drug-loaded scaffolds have emerged as a promising alternative for oral cancer treatment.

#### 2.3.8. Caries Prevention

In addition to the aspects mentioned earlier, some studies on electrospun nanomaterials have also been reported in caries prevention. Dental caries, one of the most common polymicrobial oral diseases worldwide, has received intense attention [139,140]. Garcinia mangostana (GM) extract incorporated electrospun chitosan and thiolated chitosan (TCS) nanofiber mats were employed to prevent dental caries by adhering to the buccal mucosa, the antibacterial mats could reduce oral bacteria in the oral cavity without cytotoxicity [61]. In addition to using antibacterial fiber membranes to prevent dental caries, electrospun nanofibers can also be fabricated into pH-responsive sensors for early-onset caries monitoring, the bromocresol green-polystyrene/polyvinylpyrrolidone (BCG–PS/PVP) fibrous membrane can be used to evaluate the cariogenic risk of clinical dental plaque samples by visualizing the critical pH point of 5.5, which value can induce tooth demineralization [17].

## 3. Data Sources and Methods

All the data in this paper were obtained from the Web of Science Core Collection. The eight keywords are composed of two parts: A and B. A stands for different expressions of dentistry, including “dentistry”, “dental”, “oral”, and “periodontal”, whilst B stands for different expressions of electrospinning, including “electrospinning” and “electrospun”. The publication “topic” field (i.e., title, abstract, and keywords) was searched by the above keywords on 4 February 2022. Here we refined the search results by publication years (excluding 2022), document types (including articles, processing papers, early access, and meeting abstracts) and study fields (excluding the documents about oral transmucosal drug delivery for non-oral diseases treatment), 378 publications were included in this study. The full record (including publication years, document types, authors, affiliations, publication titles, countries/regions, and the publishers) and cited references were exported to plain text file. Microsoft Excel was used to collect and analyze the data exported from the Web of Science. Origin, VOSviewer and Carrot^2^ were used to process, analyze, and evaluate the data. In particular, VOSviewer was used for the national/regional, institutional, and author cooperation network mapping and bibliographic coupling cluster mapping. The foamtree visualization with non-rectangular treemap layouts was conducted by using Carrot^2^, a website-based analyzing tool. The visualization of the distributions between each representative electrospun dental material and their applications was undertaken using the R package [141]. In our study, “China” refers to Mainland China only. Hong Kong, Macao, Taiwan, England, Scotland, Wales, and Northern Ireland are considered independent entities (“regions”) as counted by Web of Science.

## 4. Results and Discussion

In this study, a total of 378 publications were obtained according to the inclusion criteria. These publications were distributed in 161 journals, the 1949 authors come from 523 institutes in 45 countries/regions. The detailed research results are as follows.

### 4.1. Publication and Trend Analysis

**Figure 2:**
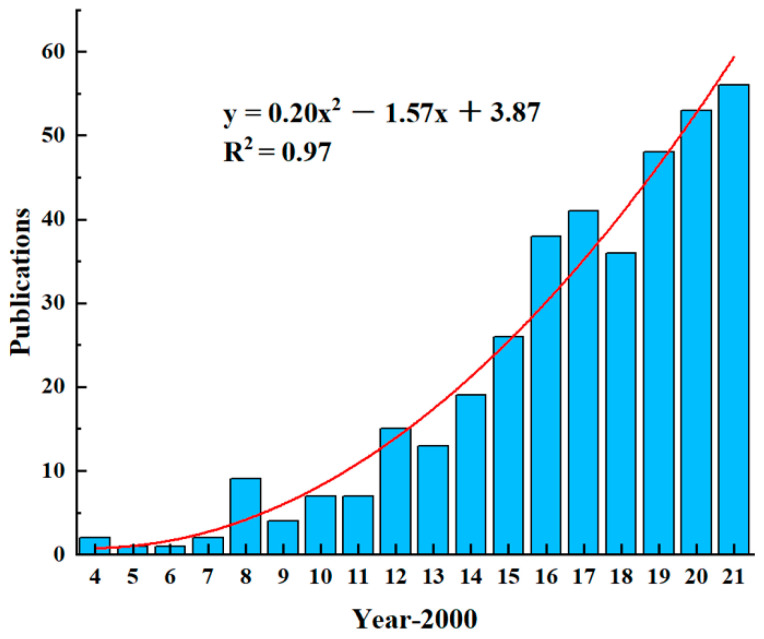
The publication output trend of electrospun dental materials from 2004 to 2021.

The total publication trend of the electrospun dental materials is demonstrated in Figure 2. The first publication can be dated back to 2004. From 2004 to 2021, the general publication trend has been upwards, especially after the year of 2011. It can be seen as an increased focus on electrospun materials’ role in dentistry. The regression analysis of the number of publications shows that y = 0.20x^2^ − 1.57x + 3.87 and the correlation coefficient R^2^ = 0.97, which means that the fitting regression effect is very good. It can be predicted that the research on electrospun dental materials will receive more attention in the next few years, and the number of publications will keep growing. The predicted publication throughout the year 2022 is 66.

### 4.2. Journal Analysis

The 378 publications were distributed in 161 journals in this study. According to the Bradford’s Law, the journals were separated into three parts with a ratio of 15:36:110. The fifteen core journals on electrospun dental materials are listed in Table 2. Nine journals belong to the first quartile (Q1), and five belongs to the second quartile (Q2), with impact factor (IF) range from 2 to 13, implying that the quality of these journals is very high. These top 15 core journals contributed 33.60% of the total publications, and the Materials Science & Engineering C-Materials for Biological Applications was the most productive journal. The topics of these journals were mainly in biomaterials, biomedical, polymer science, pharmacology and pharmacy, dentistry, oral surgery and medicine, and organic.

**Figure 3:**
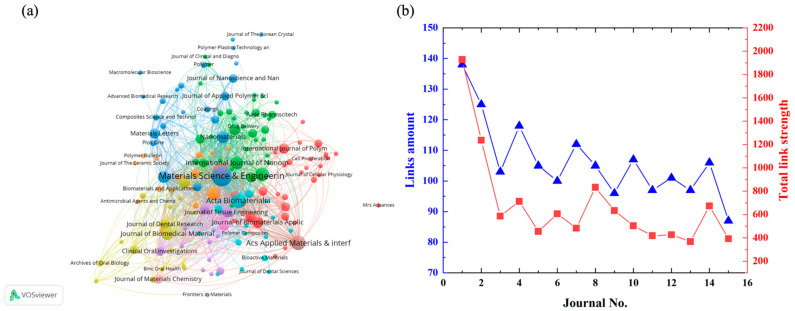
(**a**) Bibliographic coupling cluster of the core journals, (**b**) the link amount, and the total link strength among the core journals.

As shown in Figure 3a, the bibliographic coupling cluster result shows that the Materials Science & Engineering C-Materials for Biological Applications has the biggest links (138) and total link strength (1920), which means that the documents published in this journal jointly cite one or more of the same articles with the other documents published in another 138 journals, and the number of references in common was 1920. Acta Biomaterialia ranked at the second place with 125 links and 1237 total link strength. The links of most other core journals ranging from 80 and 100 while the total link strength was distributed between 800 and 1400 (Figure 3b).

### 4.3. Contribution of Country/Region

In this analysis, 45 countries/regions contributed to the publications and citations. The publication amount, ratio, h-index, total citation, and average citation of the top ten countries/regions are illustrated in Table 3. China the highest publications with 91 documents representing 17.60% of overall publications, followed by USA (70; 13.54%), Iran (42; 8.12%), England (34; 6.58%), and South Korea (32; 6.19%). Besides publication count, China also has the best performance in h-index and total citation. In comparison, South Korea was the fifth for the publications but was ranked the 3rd with an h-index of 18 and has the highest average citation.

**Figure 4:**
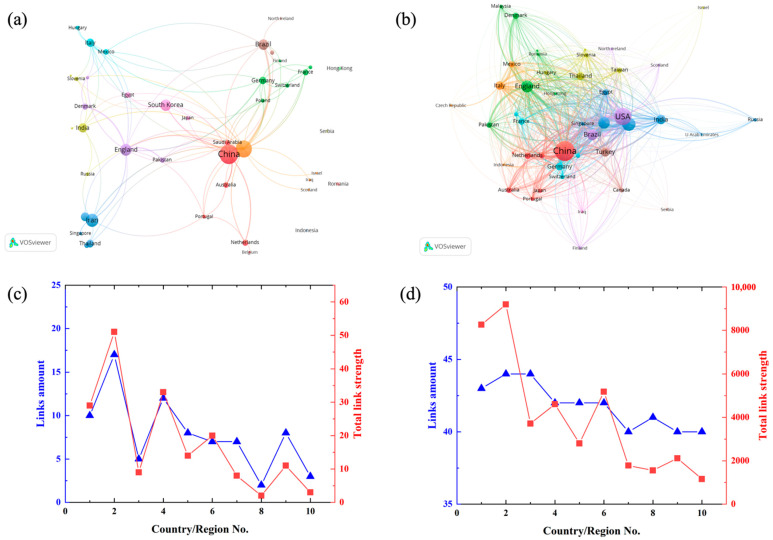
Cooperation network map and bibliographic coupling cluster of the top ten countries/regions of publications about electrospun dental materials: (**a**) National/regional cooperation network map and (**c**) the link amount, and the total link strength; (**b**) national/regional bibliographic coupling cluster, and (**d**) the link amount, and the total link strength.

Figure 4a highlights the cooperation between different countries or regions by cluster map, the bigger circle means a higher research output. Besides, the amount of lines means the cooperation between different countries/regions, and diameter of the lines means the number of the cooperation. USA has the biggest total link strength of 59 with 24 links, followed by England (36; 14), China (31; 12), and Brazil (22; 9). Countries/regions with a large number of publications tend to have more cooperation, such as USA and China (link strength of 11), USA and Brazil (link strength of 13), there are so much cooperation between them (Figure 4c). The bibliographic coupling cluster (Figure 4d). illustrates that the productive country/region also has high link strength (Figure 4b). The reference of the documents published by the top ten countries/regions show a worldwide distribution, with a links amount between 40 and 44, which means the authors of their reference from different countries/regions as illustrated in Figure 4b. The total bibliographic coupling strength of the USA (9190) and China (8262) is much higher than that of other countries/regions, which is also related to their larger number of publications.

### 4.4. Contribution of Institution

In addition to studying national or regional output and cooperation, we also analyzed research contributions between different institutions. There are 523 institutes that contributed to the electrospun dental materials publications, Table 4 shows the institutes with more than seven publications. China has five productive institutes, the USA, Thailand and Iran have two, whereas other countries/regions have only one. Among all the research institutes, University of Sheffield, with a total publication of 19 and h-index of 11, was the highest ranked research institute, followed by Indiana University (18; 15), Seoul National University (11; 9), Sichuan University (11; 7), and University of Michigan (11; 9). Regarding citation data, Seoul National University has the highest total citation and average citation (793; 72.09), followed by Radboud University (573; 71.63), and Queensland University of Technology (420; 60.00), in addition, the average citations from other institutes were less than 40. Although the total citation of Indiana University and University of Sheffield were high, their average citation were not very high. Besides, some institutes, such as University of Michigan and Tabriz University of Medical Sciences, have high total publications and h-index but low citations, meaning that the publications’ influence need to be further improved.

**Figure 5:**
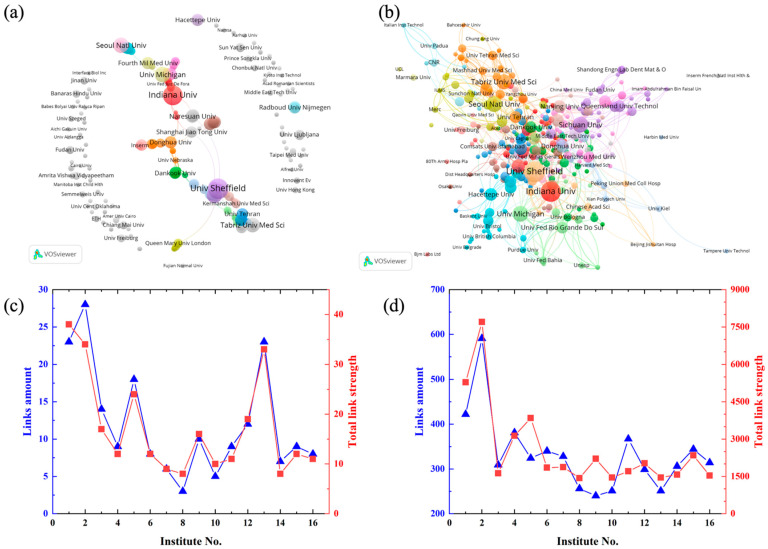
Cooperation network map and bibliographic coupling cluster of the top institutes of publications about electrospun dental materials: (**a**) institutional cooperation network map and (**c**) the link amount, and the total link strength; (**b**) Institutional bibliographic coupling relationship, and (**d**) the link amount, and the total link strength. (The minimum publications in (**c**,**d**) is 7).

The institutional research cooperation in electrospun dental materials field can be seen from the Figure 5a,c, Indiana University have the greatest cooperation relationship over the world with links of 28 and total link strength of 34, followed by University of Sheffield (23, 38) and Shahid Beheshti University of Medical Sciences (23, 33). Judging from the number of publications and cooperation, institutes with more publications tend to have more partnerships. For the bibliographic coupling (as shown in Figure 5b,d), compared with other institutions, Indiana University also shows absolute advantages with 591 links and 7699 total link strength, except that the University of Sheffield (England) has 422 links and 5280 coupling strengths. The links of other institutes were between 240 to 400, and the total link strength between 1400 to 3900.

### 4.5. Contribution of Author

**Figure 6:**
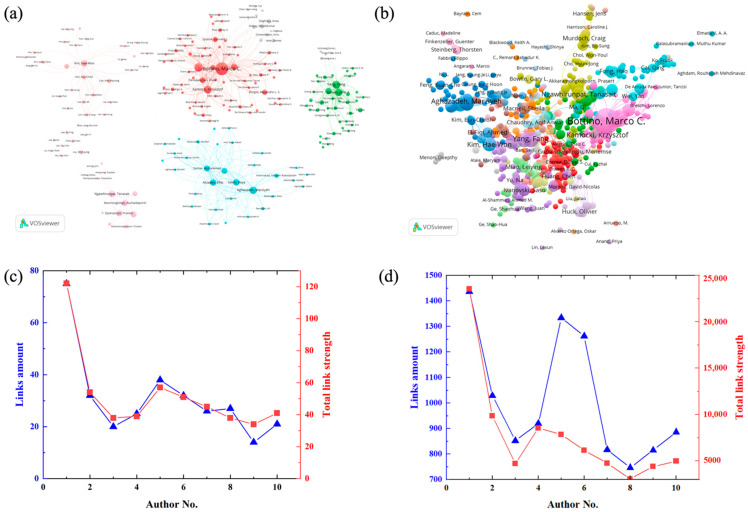
Cooperation network map and bibliographic coupling cluster of the top ten productive authors of publications about electrospun dental materials: (**a**) Cooperation network map and (**c**) the link amount, total link strength; (**b**) Bibliographic coupling cluster of all authors; and (**d**) the link amount, total link strength of the top ten authors (The minimum publications in (**a**,**c**,**d**) is 7).

A total of 1949 authors contributed to the included 378 articles. There are 28 authors who have more than five publications about electrospun dental materials. The 10 most productive authors are reported in Table 5 Marco C. Bottino was the most productive author whose publication count is twice to thrice of the number of publications than the other nine authors, with 24 research papers and an h-index of 15, followed by Gregory Richard L. (11; 10), Aghazadeh Marziyeh (9; 8), Kamocki Krzysztof (9; 9) and Yang Fang (9; 9). Apart from this, Marco C. Bottino was also the most cited researcher with a total citation of 741, and the average citation is 30.88. For Yang Fang and Jansen John A., they have very high total citation and average citation, although their publication amount is not very high, meaning that their articles are influential. The cooperation network between authors with more than seven publications is illustrated in Figure 6a,c. The analysis revealed that Marco C. Bottino has the greatest cooperation network with 75 links and the total link strength was 122. The link number of other authors was distributed from 14 to 38 and the total strength from 34 to 57. The bibliographic coupling cluster shows that Marco C. Bottino has the greatest links amount (1437), Except for Yang Fang, Jansen John A., and Gregory Richard L, which have 1334, 1262, and 1029 links, respectively, the other top ten authors have links between 700 and 950. As for the bibliographic coupling strength (23, 560), Marco C. Bottino has the greatest coupling strength which is twice to eight times those of the other nine authors.

### 4.6. Keywords Analysis

**Figure 7:**
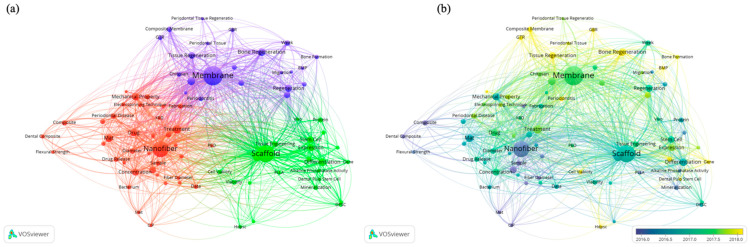
(**a**) Keywords co-occurrence clustering map of electrospun dental nanofibers; (**b**) Keywords time overlay clustering map of electrospun dental nanofibers.

Keywords are the embodiment of the main research aims and objectives of an article. Therefore, a clustering analysis of keywords can reflect the hot topics and the research prospect. It can be seen from Figure 7a that the keywords such as “nanofiber”, “membrane”, and “scaffold” have high frequency. They represent three different dimensions of electrospun dental nanomaterials. In addition, from this figure, we can see the research hotspots of “electrospun dental materials” are “Bone Regeneration”, “Tissue Regeneration”, “Cell Differentiation and Proliferation”, and “Drug Delivery”, and so on. It can also be seen that the investigation of nanofiber often involves a lot of the preparation process and the characterization of properties. In contrast, research on membranes and scaffolds have focused more on applications, such as “Human Dental Pulp Stem Cell Differentiation”, “Periodontal Regeneration”, and “Bone Formation”. Combined with the keyword time overlay clustering map in Figure 7b, the different colors referred to different average publish years, it can be seen the research direction is shifting from the nanofiber fabrication and property investigation to the application of membrane and scaffold in tissue and bone regeneration.

### 4.7. Materials and Applications Analysis

**Figure 8:**
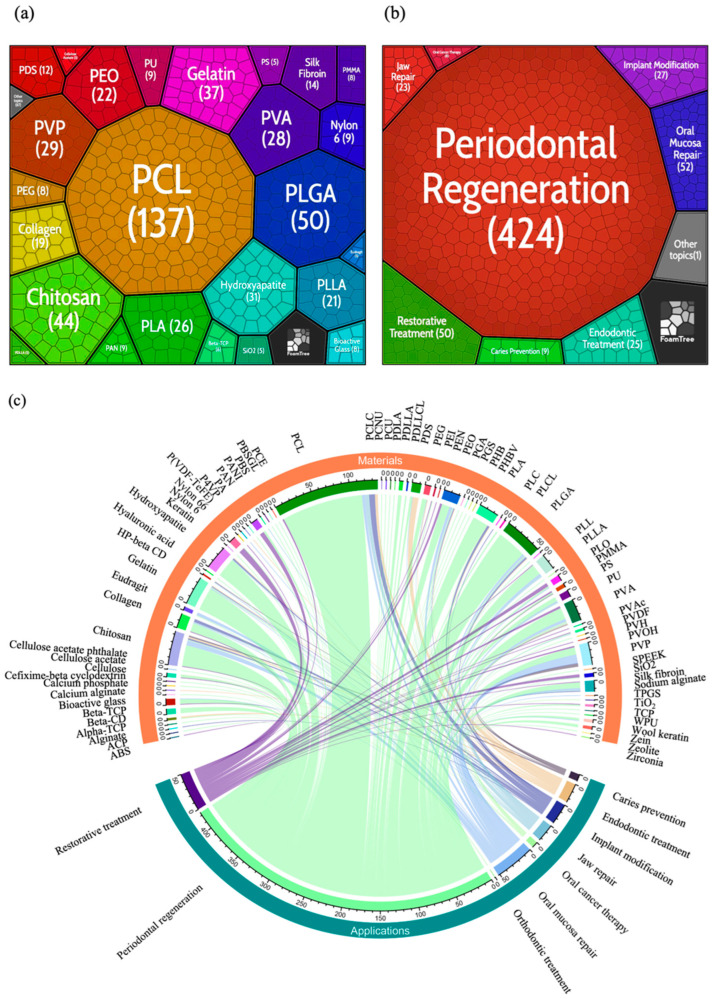
(**a**) FoamTree visualization based on representative electrospun dental materials; (**b**) FoamTree visualization based on the applications of electrospun dental nanofibers; (**c**) Chord diagram between the electrospun dental nanofibers and the applications. (The full spellings of the abbreviations in Figure 8 were shown in the supplementary materials Table S1).

To more fully interpret cutting-edge representative electrospun dental materials and their application in dentistry, the 378 publications were visualized using Carrot^2^ (Figure 8a,b) and a R package. The areas of clusters were based on the number of materials in the publications. Seventy-six kinds of materials were used 619 times in the publications. The results demonstrated that there are 10 materials used more than 20 times. As a synthetic biomaterial with good biocompatibility, biodegradability, and compatibility, PCL was the most common material in this analysis with 137 times, and PLGA, chitosan, gelatin, hydroxyapatite, PVP, PVA, PLA, PEO, and PLLA were also frequently used materials with the occurrence number between 20 to 50. For the applications, what is striking in this figure is the dominance of studies focused on the periodontal regeneration which is as many as 68.50% of those investigated, and other applications account for less than 10%. 

In the chord diagram (Figure 8c), different kinds of electrospun nanofibers for dentistry located at the top and the applied fields located at the bottom of the diagram. The width of each line is determined by the number of materials known to interact with each application. Among the materials used for periodontal regeneration, it can be seen from the chord diagram that the PCL covered 26.65% with a number of 113, followed by PLGA (41; 9.67%), gelatin (32; 7.55%), chitosan (29; 6.84%), hydroxyapatite (27; 6.37%), and PLA (21; 4.95). For other applications, the material is widely sourced and distributed evenly, for PVP applied in oral mucosa repair is more than 20% which is 23.08%. In restorative treatment, PAN and Nylon 6 are two materials that are often used. PCL is the most used material in implant modification and jaw repair while PDS is the most used material in endodontic treatment. As to caries prevention, PVP appears four times in nine applications. In oral cancer treatment, only PVA was used twice, and all the other six materials were used only once.

### 4.8. Limitations and Trends Outlooks

The limitation of this bibliometric analysis study was only documents from the WOS database were included. Secondly, the bibliometric analysis comes from the published literature, and there is a certain delay in reflecting the latest research.

In recent years, electrospun material has received much interest in dentistry because of its multifunctional properties. The increase in the number of papers published on electrospinning dental materials reflects the research level and development of the discipline to a certain extent. Through regression analysis, it is predicted that the literature will continue to grow in 2022. In the future, the research on electrospun dental materials will continue to attract the attention of researchers, and the research will be more in-depth, especially in periodontal treatment.

## 5. Conclusions

This study aims to provide a detailed analysis of electrospun dental nanofibers in the past 18 years by the bibliometric method. Our key finding is that the continuous growth in research publications indicates that electrospun dental nanofibers are receiving more attention. The top 15 core journals have contributed 33.60% of the total publications, and the Materials Science & Engineering C-Materials for Biological Applications is the most productive journal. For the contribution of the countries/regions, China has the most publications and h-index. USA, Iran, England, and South Korea have shown great performance, but there remains a gap between China. At the institute level, the University of Sheffield and Indiana University have presented significant advantages in publication amount, while Seoul National University and Radboud University have the higher total citation and average citation. Bottino Marco C. is the most prolific author with the highest publication amount and h-index. However, Jansen John A. and Yang Fang have lower publication, h-index, and total citation but higher average citations. The keywords frequency analysis presents that the hot topics in applied research are bone regeneration, tissue regeneration, cell differentiation and proliferation, and drug delivery. Furthermore, keywords analysis of research publications can help us understand the transition of the research trends, which has shifted to tissue engineering from the fabrication studies. Research on electrospun dental materials and applications shows that PCL is the most frequently used material, while most studies have focused on periodontal regeneration.

## Figures and Tables

**Table 1:** Representative Materials used in Electrospinning for Dentistry.

Material	Representative Applications	References
Natural polymer		
Alginate (AL)	Periodontal regeneration	[59]
Cellulose (CE)	Periodontal regeneration	[60]
Chitosan (CS)	Caries prevention	[61]
Collagen (CO)	Restorative treatment	[62]
Gelatin (GEL)	Jaw repair	[63]
Silk fibroin (SF)	Implant modification	[64]
Synthetic polymer		
Polyamide 6 (Nylon 6)	Restorative treatment	[26]
Polycaprolactone (PCL)	Endodontic treatment	[65]
Polyethylene oxide (PEO)	Periodontal regeneration	[66]
Polylactic acid (PLA)	Periodontal regeneration	[67]
Poly L-lactic acid (PLLA)	Endodontic treatment	[68]
Poly l-lactide-co-d, l-lactide (PDLLA)	Periodontal regeneration	[69]
Poly lactic-*co*-glycolic acid (PLGA)	Oral mucosa regeneration	[70]
Polyvinyl alcohol (PVA)	Oral cancer therapy	[71]
Polyvinylpyrrolidone (PVP)	Caries prevention	[17]
Other Nanomaterials		
β-tricalcium phosphate (β-TCP)	Periodontal regeneration	[72]
Bioactive glass (BG)	Endodontic treatment	[73]
Hydroxyapatite (HA)	Periodontal regeneration	[74]
Silicon oxide (SiO_2_)	Restorative treatment	[75]

**Table 2:** Core journals where articles on electrospun dental materials are published.

No.	Journal	TP *	IF	JIF Quartile	JIF Rank	Category
1	Materials Science & Engineering C-Materials for Biological Applications	20	7.328	Q1	7/41	Materials science, Biomaterials
2	Acta Biomaterialia	11	8.947	Q1	5/41	Materials science, Biomaterials
3	Journal of Biomedical Materials Research Part A	11	4.396	Q2	25/89	Engineering, Biomedical
4	ACS Applied Materials & Interfaces	10	9.229	Q1	44/334	Materials science, Multidisciplinary
5	Polymers	9	4.329	Q1	18/90	Polymer science
6	Biomedical Materials	8	3.715	Q2	39/89	Engineering, Biomedical
7	International Journal of Nanomedicine	8	3.715	Q1	28/276	Pharmacology & Pharmacy
8	Dental Materials	7	5.304	Q1	8/92	Dentistry, Oral Surgery & Medicine
9	Journal of Biomedical Materials Research Part B-Applied Biomaterials	7	3.368	Q2	43/89	Engineering, Biomedical
10	Nanomaterials	7	5.076	Q2	103/334	Materials science, Multidisciplinary
11	Biomaterials	6	12.479	Q1	1/41	Materials science, Biomaterials
12	Carbohydrate Polymers	6	9.381	Q1	1/63	Chemistry, Organic
13	Journal of Biomaterials Applications	6	2.646	Q3	63/106	Engineering, Biomedical
14	Materials	6	3.623	Q2	152/334	Materials science, Multidisciplinary
15	Journal of Dental Research	5	6.116	Q1	5/92	Dentistry, Oral Surgery & Medicine

* TP means total publications.

**Table 3:** Top ten countries/regions of publications about electrospun dental materials.

No.	Country/Region	TP	Ratio %	h-Index	TC *	AC *
1	China	91	17.60	28	2648	29.10
2	USA	70	13.54	24	1954	27.91
3	Iran	42	8.12	13	748	17.81
4	England	34	6.58	17	906	26.65
5	South Korea	32	6.19	18	1636	51.13
6	Brazil	28	5.42	13	432	15.43
7	India	20	3.87	9	337	16.85
8	Turkey	19	3.68	10	299	15.74
9	Italy	17	3.29	11	255	15.00
10	Thailand	15	2.90	7	267	17.80

* TC means the total citations, and AC means the average citations.

**Table 4:** Institutes with the publications about electrospun dental materials more than seven.

No.	Institutes	TP	h-Index	TC	AC
1	University of Sheffield (England)	19	11	518	27.26
2	Indiana University (USA)	18	15	626	34.78
3	Seoul National University (South Korea)	11	9	793	72.09
4	Sichuan University (China)	11	7	442	40.18
5	University of Michigan (USA)	11	9	145	13.18
6	Tabriz University of Medical Sciences (Iran)	10	8	154	15.40
7	Radboud University (Netherlands)	8	8	573	71.63
8	Naresuan University (Thailand)	8	6	222	27.75
9	Queensland University of Technology (Australia)	7	7	420	60.00
10	Silpakorn University (Thailand)	7	5	179	25.57
11	Fourth Military Medical University (China)	7	7	157	22.43
12	Soochow University (China)	7	4	145	20.71
13	Shahid Beheshti University of Medical Sciences (Iran)	7	6	129	18.43
14	Nanjing University (China)	7	5	105	15.00
15	Donghua University (China)	7	5	98	14.00
16	Hacettepe University (Turkey)	7	5	80	11.43

**Table 5:** Top ten authors of publications about electrospun dental materials.

No.	Authors	TP	h-Index	TC	AC
1	Bottino, Marco C.	24	15	741	30.88
2	Gregory, Richard L.	11	10	398	36.18
3	Aghazadeh, Marziyeh	9	8	133	14.78
4	Kamocki, Krzysztof	9	9	368	40.89
5	Yang, Fang	9	9	599	66.56
6	Jansen, John A.	8	8	584	73.00
7	Alizadeh, Effat	7	6	110	15.71
8	Kim, Hae-Won	7	6	206	29.43
9	Ngawhirunpat, Tanasait	7	5	179	25.57
10	Salehi, Roya	7	6	100	14.29

## Data Availability

Data available by corresponding author on request.

## Supplementary Materials

The following supporting information can be downloaded at: https://www.mdpi.com/article/10.3390/jfb13030090/s1, Table S1: Full spellings of the abbreviations in Figure 8.
