# Supplementary material for: A Bibliometric Analysis of Electrospun Nanofibers for Dentistry
Source: J Funct Biomater. 2022 Jul 9;13(3):90. doi: 10.3390/jfb13030090 (PMC9326643; doi:10.3390/jfb13030090)
Supplement: Supplementary file 1 [file jfb-13-00090-s001.zip › jfb-1773695-supplementary.pdf]

Table S1. Full spellings of the Abbreviations in Figure 8.

Full spellings	Abbreviations
Acrylonitrile–butadiene–styrene	ABS
Amorphous calcium phosphate	ACP
Alpha-tricalcium phosphate	Alpha-TCP
Beta-cyclodextrin	Beta-CD
Beta-tricalcium phosphate	Beta-TCP
Hydroxypropyl-β-cyclodextrin	HP beta CD
Polyamide 6	Nylon 6
Polyamide 6	Nylon 66
Poly (vinylidene fluoride-tetrafluoroethylene)	P(VDF-TeFE)
Poly(4-vinylpyridine)	P4VP
Polyamide	PA
Polyacrylonitrile	PAN
Polyaniline	PANI
Polybutylene succinate	PBS
Poly (butylene succinate-co- glycolate)	PBSGL
Polycarboxylate	PCE
Polycaprolactone	PCL
Polycarbonate polyurethane	PCNU
Polycarbonate urethanes	PCU
Poly D-lactic acid	PDLA
Poly l-lactide-co-d, l-lactide	PDLLA
Poly (D, L-lactide-co-epsilon-caprolactone)	PDLLCL
Polydioxanone	PDS
Polyethylene glycol	PEG
Polyetherimide	PEI
Poly (ethylene 2,6 naphthalate)	PEN
Polyethylene oxide	PEO
Polyglycolic acid	PGA
Poly (glycerol-sebacate)	PGS
Poly (3-hydroxybutyrate)	PHB
Poly (3-hydroxybutyrate-co-3-hydroxyvalerate)	PHBV
Polylactic acid	PLA
Poly (l-lactide-co-ε-caprolactone)	PLC
Poly (l-lactic acid-co-ε-caprolactone)	PLCL
Poly (lactic-co-glycolic acid)	PLGA

Poly (lactic acid-co-lysine)	PLL
Poly L-lactic acid	PLLA
Poly-l-Ornithine	PLO
Polymethyl methacrylate	PMMA
Polystyrene	PS
Polyurethane	PU
Polyvinyl alcohol	PVA
Polyvinyl acetate	PVAc
Polyvinylidene difluoride	PVDF
Poly (lactic acid)/Hybrid	PVH
Poly (vinyl alcohol)	PVOH
Polyvinylpyrrolidone	PVP
Silicon dioxide	SiO_2
Sulfonated poly (ether ether ketone)	SPEEK
Titanium dioxide	TiO_2
D-α-Tocopherol polyethylene glycol succinate	TPGS
Tricalcium phosphate	TCP
Water Polyurethane	WPU
